# *Tsukiyamaia*, a new genus of the tribe Baorini (Lepidoptera, Hesperiidae, Hesperiinae)

**DOI:** 10.3897/zookeys.555.6144

**Published:** 2016-01-20

**Authors:** Jian-Qing Zhu, Hideyuki Chiba, Li-Wei Wu

**Affiliations:** 1 Shanghai Zoological Park, 2381, Hongqiao Road, Shanghai, 200335, P.R. China; 2 B. P. Bishop Museum, 1525 Bernice Street, Honolulu, Hawaii, 96817-0916 U.S.A.; 3 The Experimental Forest, College of Bio-Resources and Agriculture, National Taiwan University, Nantou, Taiwan, R.O.C.

**Keywords:** Polytremis, new species, *cox1*, *cox2*, *EF-1*α

## Abstract

Skippers of the tribe Baorini are evidently a monophyletic group in the subfamily Hesperiinae. In this study, a new Baorini member *Tsukiyamaia albimacula* **gen. n.** et **sp. n.** is described from north Myanmar, southwest China and north Vietnam. Despite its peculiar and striking wing-pattern, this new genus has some important characters of Baorini, such as a broad and bifid uncus and a well-developed gnathos. Based on an analysis of male genitalia and the molecular phylogenies inferred from both mitochondrial and nuclear genes (28 taxa, total aligned length: 2968 bp), it is proposed that the genus *Tsukiyamaia* is closely related to the genus *Polytremis*, which has high species diversity in China. This study not only describes a new skipper but also highlights that *Tsukiyamaia* is important in clarifying phylogenetic relationship of *Polytremis* and its allies.

## Introduction

Phylogenetic relationships and higher classifications of Hesperiidae at tribal level were primarily settled by Warren et al. (2008, 2009) based on morphological and molecular evidence. In this phylogenetic framework, the tribe Baorini is a well-supported monophyletic group belonging to the subfamily Hesperiinae (Warren et al. 2008). This tribe was established by Doherty (1886) as Baorinae and is currently composed of eleven genera: *Brusa* Evans, 1937, *Zenonia* Evans, 1935, *Gegenes* Hübner, 1819, *Parnara* Moore, 1881, *Borbo* Evans, 1949, *Pelopidas* Walker, 1870, *Polytremis* Mabille, 1904, *Baoris* Moore, 1881, *Caltoris* Swinhoe, 1893, *Iton* de Nicéville, 1895 and *Prusiana* Evans, 1937 (Warren et al. 2009) and 99 valid species (Evans 1937, Evans 1949, Chiba and Eliot 1991, Koiwaya 1996, Tsukiyama et al. 1997, Huang 1999, Sugiyama 1999, Devyatkin and Monastyrskii 2002, Huang 2003, Vane-Wright and de Jong 2003, Yuan et al. 2010, Zhu et al. 2012). Two genera, *Brusa* and *Zenonia* are endemic to the Ethiopian region, and the other nine genera are mainly Indo-Australian and south Palaearctic (Mediterranean and Manchurian). Evans (1937, 1949) completed the most recent revision of the world’s fauna of Hesperiidae, and arranged phenotypically similar genera into informal groups in his systematics. However, phylogenetic relationship of genera within the group is not clear. The above-mentioned genera were classified in the Gegenes-group, except for *Prusiana* which was treated as a genus in the Taractrocera-group (Evans 1949). Subsequently, the Pelopidas-group (Eliot 1978) and Gegenini (Chou 1994) were proposed based on the Malaysian and Chinese faunas respectively.

The members of the tribe Baorini are brown with small semi-hyaline white spots, except for two genera, *Zenonia* and *Prusiana*, which have extensive orange markings resembling those of Taractrocerini (Warren et al. 2009). Warren et al. (2009) stated that the male genitalia were distinctive in Evans’ Gegenes-group: a relatively broad, bifid uncus, a well-developed gnathos, and the harpe terminating in an upward-pointing, serrate hook.

Recently most of newly described Baorini taxa were discovered in the range from the south boundary of Himalayas to South China (Koiwaya 1996, Tsukiyama et al. 1997, Huang 1999, Sugiyama 1999, Huang 2003, Yuan et al. 2010, Zhu et al. 2012), where species richness and endemism are obviously higher than in other regions in East Asia (Chiba 2009). Some male specimens of an undescribed species were obtained from Myanmar, which were of uncertain taxonomic position due to only male characters. Subsequently, a female and some male specimens were added from southwest China and Vietnam, and molecular phylogenies based on mitochondrial and nuclear genes were inferred. This investigation suggests that this new species belongs to a new genus of the tribe Baorini, which is sister to *Polytremis*.

## Methods

### Sampling

For morphological comparison, eight male and one female specimens of this new taxon were examined. For inferring phylogenetic relationships of tribe Baorini to investigate the position of the new genus, 28 species were sampled (Table 1). A total of seven out of eleven genera in Evan’s Gegenes-group were sampled and they are all distributed with *Tsukiyamaia* in Indo-Australian and the south Palaearctic region. Data of ten taxa were obtained from previous studies (Warren et al. 2008, 2009; Table 1).

**Table 1.** List of the skippers used in this study.

Name	Voucher	Locality	Accession number		Reference
			*cox1-cox2*	*EF-1*α	
*Calpodes ethlius*	144-ADW		EU364494	EU364289	Warren et al. 2008
*Dubiella belpa*	458-ADW		EU364051	EU364249	Warren et al. 2008
*Ochlodes bouddha*	H1-0635	Taiwan	KT240162	KT240144	this study
*Pyrrhopygopsis crates*	64-ADW		EU364503	EU364298	Warren et al. 2008
*Saliana esperi*	514-ADW		EU364501	EU364296	Warren et al. 2008
*Suastus gremius*	H1-1548	Taiwan	KT240163	KT240145	this study
*Synapte silius*	634-ADW		EU364431	EU364226	Warren et al. 2008
*Talides sinois*	512-ADW		EU364457	EU364252	Warren et al. 2008
*Thracides phidon*	451-ADW		EU364502	EU364297	Warren et al. 2008
*Udaspes folus*	H1-1546	Taiwan	KT240164	KT240146	this study
*Baoris farri*	H1-0260	Sichuan, China	KT240165	KT240147	this study
*Brobo cinnara*	H1-0684	Fujian, China	KT240166	KT240148	this study
*Caltoris bromus*	H1-1645	Taiwan	KT240167	KT240149	this study
*Caltoris cahira*	H1-1644	Taiwan	KT240168	KT240150	this study
*Iton watsonii*	600-MCZ		EU364490	EU364285	Warren et al. 2008
*Parnara guttata*	H1-1008	Sichuan, China	KT240169	KT240151	this study
*Pelopidas conjuncta*	H1-1565	Taiwan	KT240170	KT240152	this study
*Pelopidas mathias*	H1-0617	Taiwan	KT240171	KT240153	this study
*Pelopidas thrax*	570-ADW		EU364492	EU364287	Warren et al. 2008
*Polytremis gotama*	H1-1019	Yunnan, China	KT240172	KT240154	this study
*Polytremis kiraizana*	H1-1437	Taiwan	KT240173	KT240155	this study
*Polytremis lubricans*	H1-0052	Taiwan	KT240174	KT240156	this study
*Polytremis matsuii*	H1-0982	Sichuan, China	KT240175	KT240157	this study
*Polytremis nascens*	H1-0321	Sichuan, China	KT240176	KT240158	this study
*Polytremis pellucida*	234-ADW		EU364493	EU364288	Warren et al. 2008
*Polytremis zina*	H1-0607	Taiwan	KT240177	KT240159	this study
*Pseudobrobo bevani*	H1-0888	Yunnan, China	KT240178	KT240160	this study
*Tsukiyamaia albimacula*	H1-1661	Yunnan, China	KT240179	KT240161	this study

### Morphological procedures

We employed the standard method in Lepidoptera research to examine the male and female genitalia as well as other morphological characters of *Tsukiyamaia* (Zhu et al. 2012). The terminology for wing patterns followed Evans (1949) and for genitalia Shirôzu (1960) and Ehrlich (1958).

The holotype and one female paratype of the new taxon were deposited in Department of Biology, Shanghai Normal University, China. One male paratype was deposited in the private collection of Jia-Qi Wang. The other paratype from China is in the collection of Kadoorie Conservation China, Kadoorie Farm and Botanic Garden, Hong Kong. The rest of the paratypes are in Hiroshi Tsukiyama’s collection (Chiba-pref., Japan).

### Molecular procedures

Genomic DNA was extracted from the thoracic or leg tissue via using the Purgene DNA Isolation kit (Gentra Systems, Minnesota, USA), following the manufacturer protocol. The primers used for amplifying the mitochondrial cytochrome c oxidase I and II (*cox1* and *cox2*) and nuclear elongation factor 1 alpha (*EF-1*α) genes were adopted from previous studies (Caterino and Sperling 1999; Kandul et al. 2004; Simonsen et al. 2010; Lu et al. 2009). Each PCR reaction was carried out in a final volume of 30 µL with 0.32 µM dNTP, 1.5 mM MgCl2, 0.2 µM of each primer, 1X Taq buffer, 1U Taq DNA polymerase, and finally added dH_2_O up to 30 µL. The PCR program was setting as 2 min at 94 °C, followed by 35 cycles of 30 s at 94 °C, 30 s at 50–55 °C, and 1–2 min at 72 °C. The final elongation step was continued for 7 min at 72 °C, and stopped at 4 °C. The PCR products were checked on 1.0 % agarose gels in 1X TBE buffer to ensure the PCR fragments were correctly amplified. DNA sequences were obtained by an ABI 3730 DNA Analyzer (Applied Biosystems, Foster City, CA, USA).

Molecular sequences of the *cox1*-*cox2* and *EF-1*α genes were checked and assembled into contiguous arrays using Sequencher 4.8 (GeneCode, Boston, USA). After primer regions were cropped, the sequence dataset was aligned according to amino sequence similarity with the default settings by MUSCLE (Edgar 2004) in MEGA 5.1 software package (Tamura et al. 2011). Missing data and ambiguities were designated to IUPAC codes, and all the sequences were submitted to GenBank (Assession No. KT240144-KT240179; Table 1)

To evaluate species differentiation among Baorini skippers, genetic distance between species was calculated via MEGA 5.1. Pairwise distance with Kimura-2-parameter (Kimura and Ohta 1972) was performed, and bootstrap method was used to estimate its variance. For reconstructing phylogenies, two methods were used: Bayesian inference (BI) was carried out by using MrBayes v. 3.2.1 (Ronquist et al. 2012), and Maximum Likelihood (ML) was performed in RAxML Pthreads-based SSE3 version 7.4.2 (Ott et al. 2007; Stamatakis 2006). In BI method, the substitution model was set to GTR+Г (GTR: General Time Reversible; Г: gamma distribution), and the taxa *Udaspes folus* was set as functional outgroup for investigate genus relationship among Baorini based on the latest phylogenetic relationship of skippers (Warren et al. 2009). To evaluate the effect of different partition strategies, four different datasets were executed: (1) no partition (combined dataset); (2) two gene region partitions (mitochondrial and nuclear genes); (3) four gene partitions (*cox1*, tRNA-Leu, *cox2*, and *EF-1*α), and (4) both gene and codon partition (ten partitions). Each partition matrix has its independent substitution model if partition was setting. Each dataset has run with six chains (five heated and one cold) for one million generations and sampled trees every 100 generations. The log-likelihood scores were plotted against generation time, and then burn-in the first 25% trees and the remaining trees were used for representing the posterior probability if the stationarity was reached. In ML method, datasets were processed with the non-default settings as follows: substitution model was set to GTRGAMMA. Outgroup and the four partition datasets were set as the BI method. The node support values of ML topology were evaluated by 1000 bootstrap (BS) replicates with ten additional searches per replicate to improve the confidence of each bootstrap search.

## Results

### Morphological systematics

#### Tsukiyamaia gen. n.

Taxon classificationAnimaliaLepidopteraHesperiidae

http://zoobank.org/23C5AC28-1908-4451-A2E6-3A4A8FA39CA4

Figs 1–4

##### Type species.

*Tsukiyamaia albimacula* sp. n.; designated by monotypy.

##### Description.

Antennae: 9.5−10 mm in length, half-length of forewing, nudum 13–14 on apiculus; Labial palpi: Second segment stout and erect, with brown hairs dorsally and yellowish hairs ventrally; third segment short, pointed and erect. Legs: middle tibiae unspined. Wing-shape: Forewing 19−20 mm in length, triangular in shape; costa about 1.4 times as long as dorsum, approximately straight, weakly arched on anterior half; apex angulated; termen lightly curved on anterior half; inner dorsum almost straight. Hindwing nearly triangular in shape; costa slightly longer than dorsum, obviously arched; termen curved on anterior half; tornus concave; dorsum almost straight.

**Wing venation** (Fig. 5)**.** Forewing: vein 2A very short not reaching dorsum; vein Cu_2_ arising before the origin of vein R_1_ and slightly nearer the origin of Vein M_3_ than to base; Vein M_2_ obviously closer to Vein M_3_ than to Vein M_1_ at origin; cell longer than half the wing length. Hindwing: Vein Cu_1_ arising beyond the origin of Vein M_1_; Vein Cu_2_ arising beyond the origin of Vein Rs; Vein M_2_ absent. Discocellular veins on both wings obvious.

**Figures 1–5.**
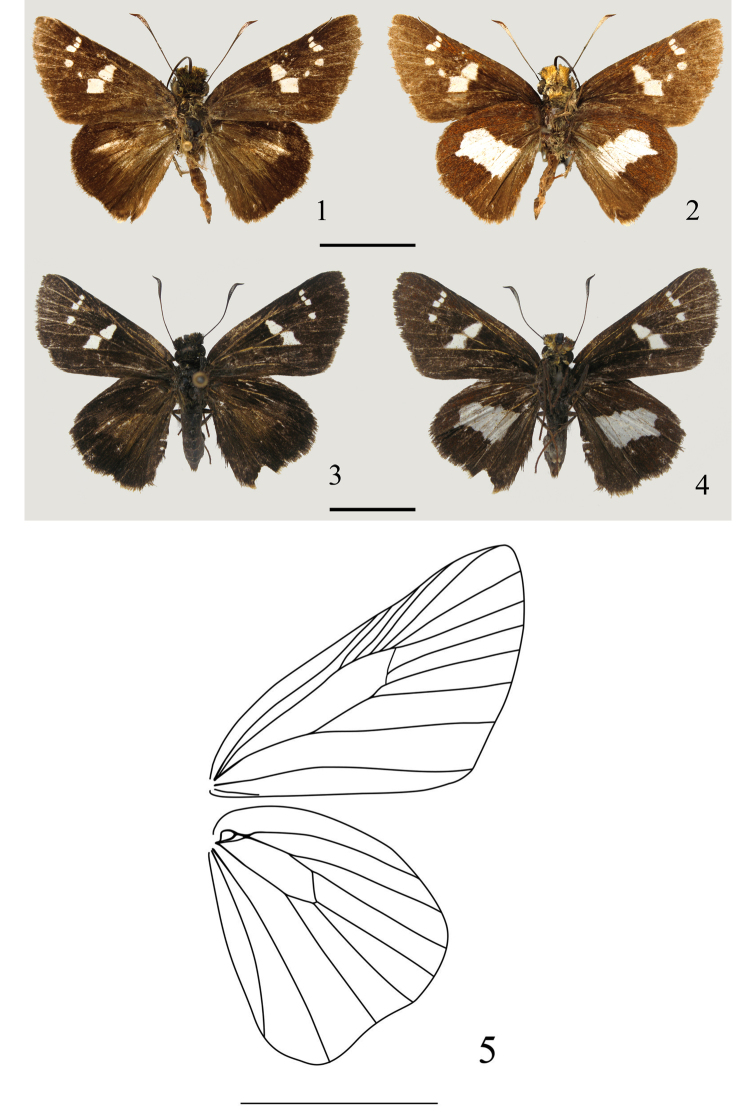
*Tsukiyamaia albimacula*. **1** holotype, ♂, upperside **2** holotype, ♂, underside **3** paratype, ♀, upperside **4** paratype, ♀, underside **5** wing venation. Scale bar: 10 mm.

**Wing markings** (Figs 1–4)**.** without stigma or secondary sexual characters; forewing with semi-hyaline spots in spaces Cu_2_, M_3_, M_2_, R_3_, R_4_, R_5_ and cell; hindwing upperside with a cigar-shaped spot in space M_2_, underside centrally with a large white marking restricted from vein 2A to vein Rs.

**Figures 6–10.**
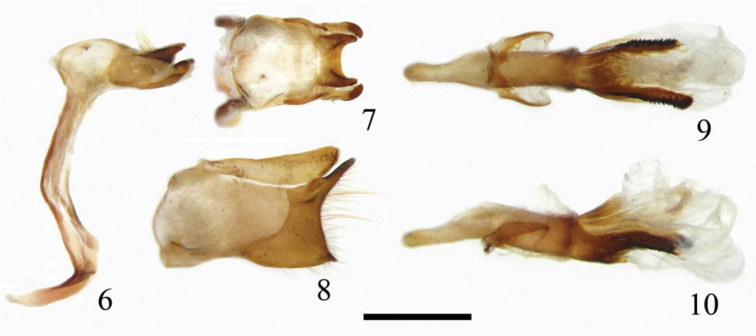
Male genitalia of *Tsukiyamaia albimacula*. **6** lateral view of ring **7** dorsal view of tegumen **8** outer view of left valva **9** ventral view of phallus **10** lateral view of phallus. Scale bar: 1 mm.

**Male genitalia** (Figs 6–10)**.** Tegumen swollen; uncus U-shaped bifurcated; gnathos bifurcated, slightly turned inside at tip and outwardly spined; valva approximately rectangle; dorsal process of harpe well produced; ventral process of harpe weakly protruded; phallus deeply bifid distally, well protruded and heavily spined outwardly; cornuti absent; manica membranous; juxta U-shaped.

##### Etymology.

The generic name is derived from Hiroshi Tsukiyama, whose outstanding contribution to the taxonomy of Hesperiidae is noteworthy.

#### Tsukiyamaia albimacula sp. n.

Taxon classificationAnimaliaLepidopteraHesperiidae

http://zoobank.org/6ED6C0E1-0571-4536-BF4B-B00F857B19FE

##### Description.

Antennae 9.5−10 mm in length, about 1/2 the length of forewing, black brown except club gray dorsally and grayish yellow ventrally; nudum 13-14 on apiculus. Palpi erect, with brown hairs dorsally and yellowish hairs ventrally. Thorax and abdomen covered with brown hairs. Forewing 19−20 mm in length. Both wings ground color black brown at each sides, with white spots and marking; costal area of forewing and entire hindwing covered with brown scales underside; cilia of both wings brown. Upperside forewing: three apical spots in spaces R_3_−R_5_, arranged linear; one discal spot present at the middle of the space M_2_; in space M_3_, a reduced spot present in the holotype, and absent in two paratypes; cell spots conjoined as trapezium-shaped, which also conjoined with the Cu_1_ spot. Underside forewing markings same as upperside. Upperside hindwing: only with a cigar-shaped spot in space M_1_. Underside hindwing: Discal area with a very large, rectangle white marking extending from vein Rs to the middle of space Cu_2_. Inward margin smooth, upward to the end of the discal cell. Outward margin lightly serrated, and evidently elongated in space M_1_.

**Male genitalia** (Figs 6–10)**.** Tegumen swollen; uncus U-shaped, bifurcated dorsally, pointed at tip laterally; gnathos bifurcated, longer and wider than uncus, slightly turned inside at tip and outwardly spined; saccus short, pointed distally; valva approximately rectangle; ampulla slightly elongate upward, harpe dorsally with a long and straight elongated process and ventrally with a relatively short and small process, outward margin concave and covered with dense hairs; costa smooth dorsally, sacculus concave ventrally; phallus 1.4 times as long as valva; subzonal about 1.3 times as the length of suprazonal, distally deeply bifid as two protruded processes, equal in length and heavily spined outwardly; without cornuti; manica membranous; juxta U-shaped.

**Figures 11–12.**
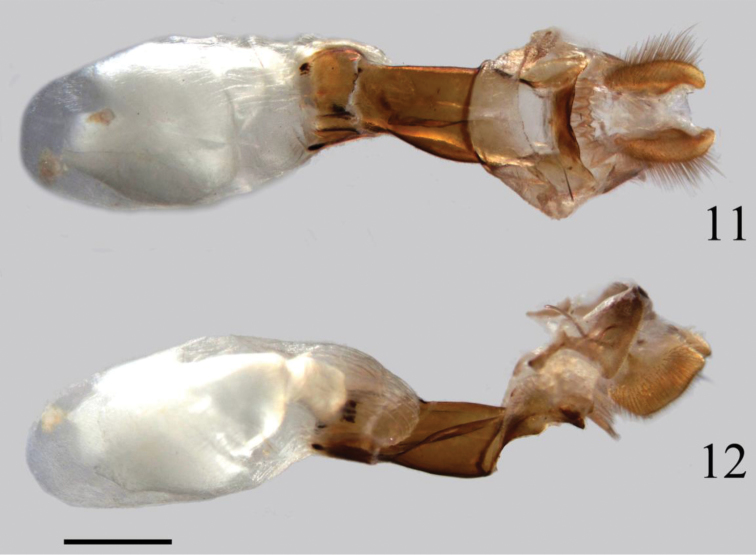
Female genitalia of *Tsukiyamaia albimacula*. **11** ventral view **12** lateral view. Scale bar: 1 mm.

**Female genitalia** (Figs 11–12)**.** Papilla analis nearly rectangle, covered with hairs on the surface; apophysis posterioris slender and short; Lamella postvaginalis oblong with outer margin arched; lamella antevaginalis with triangular parts laterally, slightly sclerotized; ductus bursae short, wide as ostium bursae, strongly sclerotized; bursa copulatrix oval, membranous with no signum.

HOLOTYPE ♂: Phutao, Kachin, N. of MYANMAR, ~1000m, 09-VI-2000, Male genitalia examined by H. Chiba, #HC030511.

PARATYPES: 1♂, the same locality as the holotype, 29-V-2000.; 1♂ ditto, 08-VI-2000.; 1♂ Panglan, ~700m, Kachin, N. of MYANMAR, 02-IX-2002. 1♂ ditto, 04-IX-2002. . 1♂ ditto, 05-IX-2002, 1♂ ditto, 29-IX-2002, 1♂ Mt. Fan Shi Pang, ~1800m, N. VIETNAM, IV-2002. 1♂ Baopo, Dulongjiang, Yunnan, CHINA, 1500m, 29-V-2011, Jia-Qi Wang leg.; 1♀ , Maku, Dulongjiang, Yunnan, CHINA, 1900m, 03-VI-2009, Jian-Qing Zhu, leg; 1♂ CHINA, Yunnan, Tengchong, Gaoligongshan National Nature Reserve, Zhengding, 2200m, 26-IV-2014, LO Yik Fui Philip coll. (YFL140055).

**Voltinism.** Judging from the collecting data, the species is expected to be multivoltine.

##### Distribution

**Figure 13.**
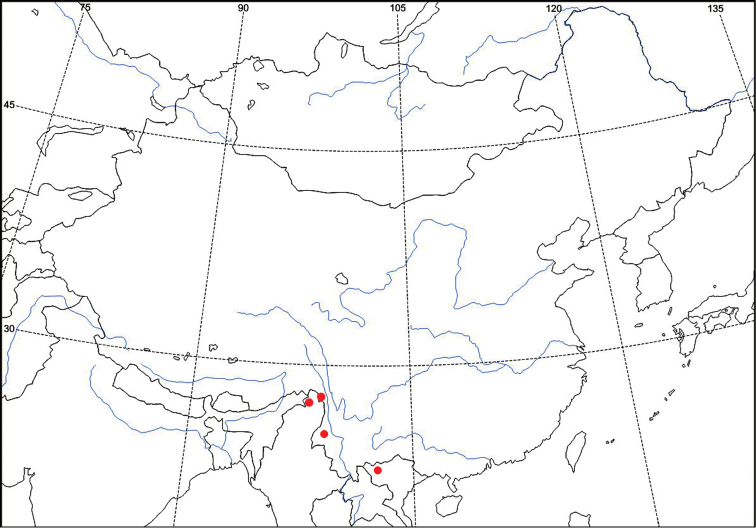
Distribution map for *Tsukiyamaia albimacula*, red circle.

(Fig. 13)**.** China (Yunnan), Myanmar (Kachin), and Vietnam (Mt. Fan Shi Pang).

##### Biology.

*Tsukiyamaia* prefers open habitats, such as open field on the hillside, farmland and heavily disturbed shrub land. It is active near the ground and stream under strong sunlight. The female frequents flowers and the male performs padding behavior.

##### Etymology.

The species is named for its large white marking on underside of the hindwing.

##### Diagnosis.

In appearance, *Tsukiyamaia* is peculiar in Baorini with a large white marking in the center of the hindwing underside. The male genitalia of *Tsukiyamaia* can be separated from those of Baorini genera by the uncus lacking a pair of basal processes, and the harpe dorsally with a long and straight elongated process and ventrally with a relatively short and small process.

### Molecular information Sequence information

The gene length used in this study included *cox1* (1531bp), tRNA-Leu (71 bp), *cox2* (141 bp), and *EF-1*α (1225 bp) genes. Pairwise distance based on mitochondrial sequences showed that the smallest one between *Tsukiyamaia albimacula* and *Polytremis matsuii* was 6.8% (Table 2). If it was compared with other *Polytremis* skippers, it ranged from 7.2 to 10.6%. Whereas comparing to other genera, it ranged from 7.6% (*Iton watsonii*) to 12.8% (*Dubiella belpa*).

**Table 2.** Pairwise distance based on the substitution model of Kimura 2-parameter and *cox1 – cox2* sequences (aligned length 1743 bp). The dash symbol means the overlap sequence is below 50 bp, the value is excluded.

	1	2	3	4	5	6	7	8	9	10	11	12	13	14
1 *Udaspes folus*														
2 *Suastus gremius*	0.106													
3 *Synapte silius*	0.122	0.101												
4 *Thracides phidon*	0.123	0.116	0.113											
5 *Pyrrhopygopsis crates*	0.137	0.105	0.116	0.130										
6 *Talides sinois*	0.121	0.065	0.097	0.109	0.118									
7 *Ochlodes bouddha*	0.124	0.104	0.095	0.113	0.116	0.097								
8 *Dubiella belpa*	0.132	0.111	0.112	0.105	0.133	0.118	0.096							
9 *Calpodes ethlius*	0.120	0.067	0.094	0.097	0.107	0.087	0.096	0.107						
10 *Saliana esperi*	0.118	0.080	0.101	0.100	0.124	0.095	0.088	0.118	0.071					
11 *Parnara guttata*	0.131	0.121	-	-	-	-	0.123	-	-	-				
12 *Baoris farri*	0.134	0.124	-	-	-	-	0.113	-	-	-	0.110			
13 *Pelopidas mathias*	0.113	0.111	0.106	0.096	0.112	0.104	0.103	0.115	0.097	0.102	0.109	0.090		
14 *Pelopidas thrax*	0.121	0.091	0.115	0.112	0.118	0.100	0.100	0.121	0.098	0.096	-	-	0.060	
15 *Pelopidas conjuncta*	0.123	0.111	0.103	0.104	0.117	0.104	0.103	0.116	0.114	0.115	0.094	0.086	0.059	0.059
16 *Brobo cinnara*	0.122	0.109	-	-	-	-	0.117	-	-	-	0.112	0.096	0.092	-
17 *Iton watsonii*	0.113	0.112	0.106	0.100	0.110	0.089	0.093	0.117	0.101	0.102	-	-	0.074	0.084
18 *Caltoris cahira*	0.126	0.098	0.111	0.115	0.127	0.109	0.106	0.117	0.101	0.101	0.116	0.117	0.101	0.100
19 *Caltoris bromus*	0.125	0.113	0.116	0.113	0.129	0.105	0.114	0.119	0.096	0.103	0.127	0.137	0.110	0.095
20 *Pseudobrobo bevani*	0.126	0.099	-	-	-	-	0.123	-	-	-	0.114	0.113	0.102	-
21 *Polytremis lubricans*	0.137	0.133	0.097	0.116	0.135	0.126	0.124	0.151	0.106	0.107	0.131	0.117	0.104	0.122
22 *Polytremis matsuii*	0.127	0.129	-	-	-	-	0.105	-	-	-	0.089	0.100	0.091	-
23 *Polytremis kiraizana*	0.123	0.116	0.107	0.103	0.128	0.101	0.103	0.115	0.109	0.102	0.104	0.107	0.094	0.092
24 *Polytremis nascens*	0.116	0.115	-	-	-	-	0.113	-	-	-	0.105	0.100	0.092	0.000
25 *Polytremis gotama*	0.107	0.115	-	-	-	-	0.104	-	-	-	0.101	0.083	0.086	0.000
26 *Polytremis zina*	0.116	0.115	0.108	0.123	0.142	0.100	0.107	0.117	0.092	0.109	0.093	0.091	0.082	0.089
27 *Polytremis pellucida*	0.117	0.124	0.115	0.116	0.123	0.096	0.107	0.116	0.104	0.108	0.099	0.095	0.083	0.089
28 *Tsukiyamaia albimacula*	0.120	0.119	0.114	0.121	0.123	0.114	0.108	0.128	0.121	0.109	0.105	0.098	0.087	0.085

	15	16	17	18	19	20	21	22	23	24	25	26	27
1 *Udaspes folus*													
2 *Suastus gremius*													
3 *Synapte silius*													
4 *Thracides phidon*													
5 *Pyrrhopygopsis crates*													
6 *Talides sinois*													
7 *Ochlodes bouddha*													
8 *Dubiella belpa*													
9 *Calpodes ethlius*													
10 *Saliana esperi*													
11 *Parnara guttata*													
12 *Baoris farri*													
13 *Pelopidas mathias*													
14 *Pelopidas thrax*													
15 *Pelopidas conjuncta*													
16 *Brobo cinnara*	0.085												
17 *Iton watsonii*	0.078	-											
18 *Caltoris cahira*	0.106	0.122	0.089										
19 *Caltoris bromus*	0.111	0.115	0.083	0.077									
20 *Pseudobrobo bevani*	0.098	0.096	-	0.109	0.116								
21 *Polytremis lubricans*	0.111	0.121	0.088	0.116	0.133	0.095							
22 *Polytremis matsuii*	0.084	0.095	-	0.119	0.124	0.107	0.105						
23 *Polytremis kiraizana*	0.087	0.084	0.072	0.105	0.112	0.098	0.105	0.061					
24 *Polytremis nascens*	0.092	0.091	-	0.102	0.102	0.095	0.098	0.065	0.077				
25 *Polytremis gotama*	0.085	0.085	-	0.102	0.094	0.088	0.089	0.061	0.066	0.038			
26 *Polytremis zina*	0.081	0.079	0.073	0.109	0.117	0.087	0.094	0.061	0.069	0.052	0.051		
27 *Polytremis pellucida*	0.081	0.083	0.075	0.113	0.121	0.095	0.104	0.060	0.073	0.056	0.057	0.009	
28 *Tsukiyamaia albimacula*	0.089	0.093	0.076	0.104	0.115	0.110	0.106	0.068	0.077	0.079	0.074	0.072	0.078

### Molecular phylogenies

**Figure 14.**
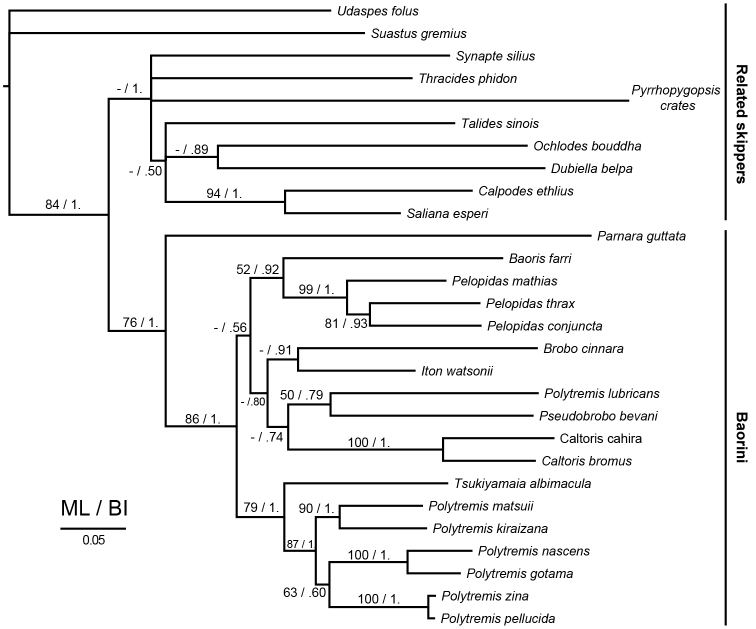
Bayesian phylogeny of the tribe Baorini based on four gene-partitioned dataset. The numbers above or below the branches are the ML bootstrap value / BI posterior probability.

The total of eight topologies, inferred by four partitioning datasets and by two tree-reconstructing methods, have similar phylogenetic relationships (summarized in Fig. 14, Appendix: S1–S3). All the Baorini members are grouped together and *Parnara guttata* is the most primitive taxa. Although the genus-level relationships within Baorini are still unresolved, *Tsukiyamaia* is sister to *Polytremis* members with high support value. In addition, our Baorini topology also indicated that the genus *Polytremis* might not be a monophyletic group, and more taxa-sampling is needed for further phylogenetic studies.

## Discussion

Although the monophyly of the tribe Baorini is well-supported by the molecular data, no synapomorphic character in external morphology have been found (Warren et al. 2009). Characters are either shared by most but not all the members of the tribe, or shared by members of other tribes.

Evans (1949) merely gives diagnostic difference between his Gegenes- group (= Baorini) and Taractrocera-group (= Taractrocerini), which is the wing color. The former is brown while the latter is yellow or orange. As mentioned in the introduction, it is not applicable for *Prusiana*, which Evans (1949) considered a member of Taractrocera-group, nor the African *Zenonia* as well as the new genus. The outstanding coloration of *Tsukiyamaia* may imply that there exist some unknown adaptive advantages driving the evolution of the peculiar marking with the slightest resemblance to its allies.

Eliot (1978) claims that the “internal veinlet entering the cell from just above the origin of vein 3 on the forewing” is the character shared by members of his Pelopidas-group but not the Taractrocera-group of genera. However, he only illustrated the wing venation of *Caltoris tulsi*, which apparently shows the veinlet. Figures of wing venation in Bascombe et al. (1999) suggest that the veinlet can be observed clearly only in *Caltoris bromus*, recognizable in *Borbo cinnara*, *Pelopidas conjunctus*, and *Polytremis lubricans*, absent in *Parnara guttata* and *Baoris farri*. We could not recognize the veinlet in the wing venation of *Tsukiyamaia*.

If the key for separation of genera in Evans (1949) or Eliot (1978) is applied, *Tsukiyamaia* is assigned to *Polytremis*, which is consent to the phylogeny based on molecular data.

## Supplementary Material

XML Treatment for Tsukiyamaia

XML Treatment for Tsukiyamaia albimacula

## Figures and Tables

**Figure S1.**
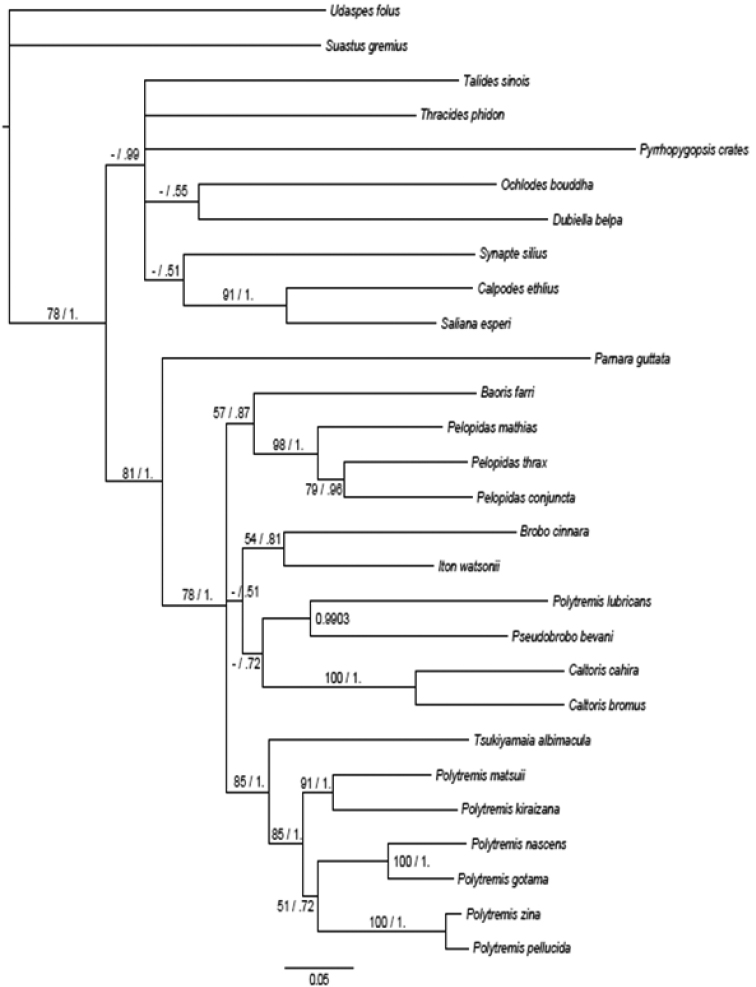
Bayesian phylogeny of the tribe Baorini based on no partitioned dataset. The numbers above or below the branches are the ML bootstrap value / BI posterior probability.

**Figure S2.**
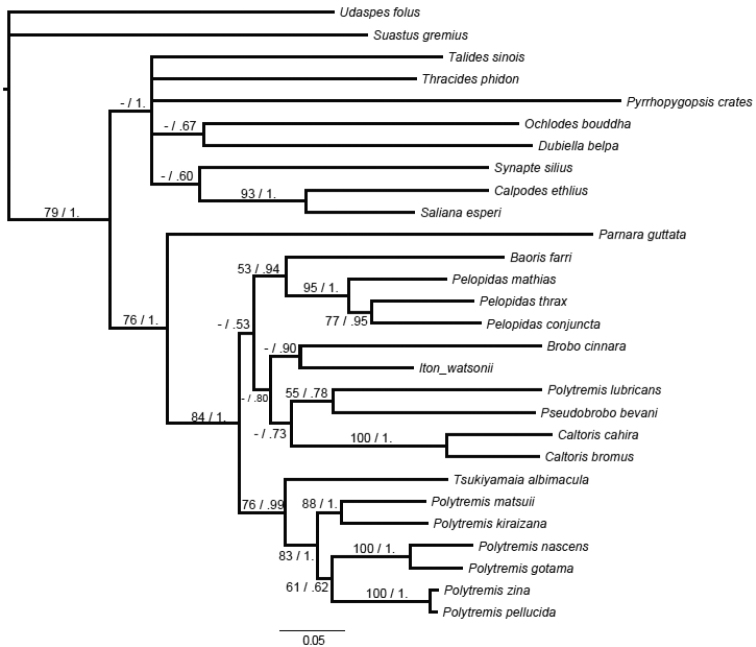
Bayesian phylogeny of the tribe Baorini based on two partitioned dataset. The numbers above or below the branches are the ML bootstrap value / BI posterior probability.

**Figure S3.**
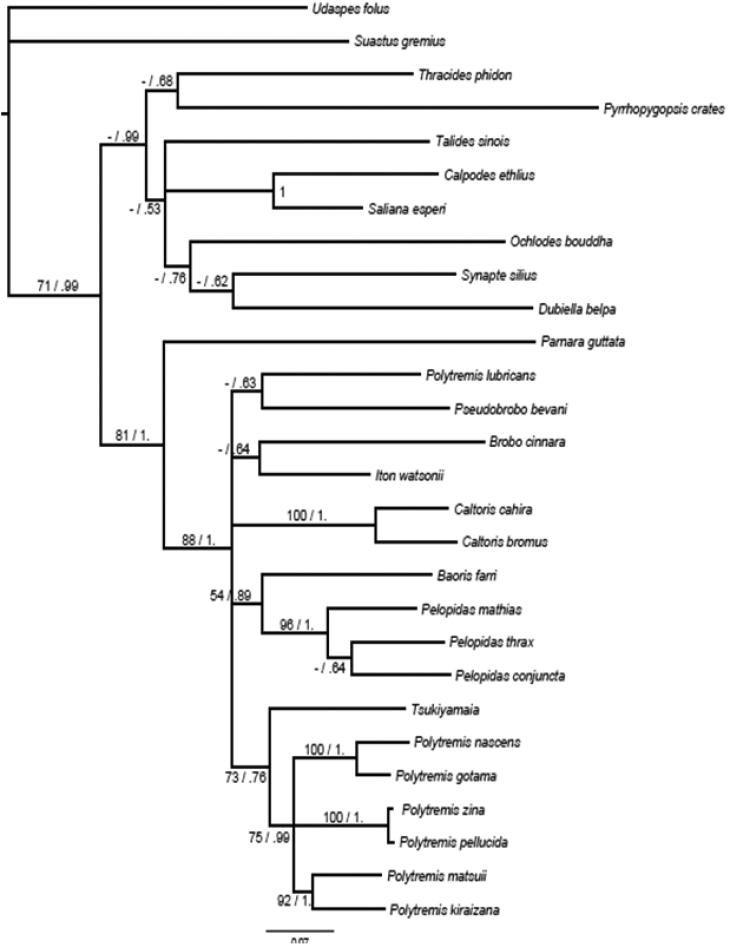
Bayesian phylogeny of the tribe Baorini based on codon partitioned dataset. The numbers above or below the branches are the ML bootstrap value / BI posterior probability.
